# Prevalence and antimicrobial resistance of *Acinetobacter baumannii* isolated from endotracheal aspirates in a tertiary-care intensive care unit from South India

**DOI:** 10.3389/fmicb.2026.1784001

**Published:** 2026-04-15

**Authors:** Deepthi Puttegowda, Lakshmi Jayaram, Deepashree Rajshekar, Stavelin Abhinandithe K., Sowjanya Pulipati, Maciej Przybyłek, Chandrashekar Srinivasa, Ramith Ramu, Mudassar Shahid

**Affiliations:** 1 Department of Biotechnology and Bioinformatics, School of Life Sciences, JSS Academy of Higher Education & Research, Mysore, Karnataka, India; 2 Department of Microbiology, JSS Medical College & Hospital, JSS Academy of Higher Education & Research, Mysore, Karnataka, India; 3 Division of Medical Statistics, School of Life sciences, JSS Academy of Higher Education & Research, Mysore, Karnataka, India; 4 Department of Pharmaceutics, Vignan Pharmacy College (Autonomous), Vadlamudi, Guntur, Andhra Pradesh, India; 5 Department of Physical Chemistry, Pharmacy Faculty, Collegium Medicum of Bydgoszcz, Nicolaus Copernicus University in Toruń, Bydgoszcz, Poland; 6 Department of Studies in Biotechnology, Davangere University, Shivagangothri, Davangere, Karnataka, India; 7 Department of Pharmaceutics, College of Pharmacy, King Saud University, Riyadh, Saudi Arabia

**Keywords:** *Acinetobacter baumannii*, carbapenem resistance, endotracheal aspirates, receiver operating characteristic analysis, ventilator-associated pneumonia

## Abstract

**Background:**

Multidrug-resistant (MDR) *Acinetobacter baumannii* is a major cause of ventilator-associated infections in intensive care units (ICUs), where empirical therapy is often initiated without up-to-date antimicrobial resistance (AMR) surveillance data. This study assessed the prevalence, demographic associations, and antimicrobial susceptibility patterns of MDR *A. baumannii* isolated from endotracheal (ET) aspirates in a tertiary-care hospital in southern India.

**Methods:**

In this single-center retrospective observational study, 3,540 non-duplicate clinical isolates collected between 2019 and 2025 were analyzed. Bacterial identification and antimicrobial susceptibility testing were performed using the Vitek 2 Compact automated system and the Kirby–Bauer disc diffusion method, with results interpreted in accordance with Clinical and Laboratory Standards Institute (CLSI) performance standards. Receiver operating characteristic (ROC) curve analysis was used to evaluate the discriminatory performance of the commonly used antibiotics, with the results expressed as the area under curve (AUC).

**Results:**

MDR *A. baumannii* accounted for 20.2% of all isolates, with the highest prevalence among neurosurgical ICU patients (22.1%). Isolates were more frequent in males (74%) and in patients aged ≥30 years. Resistance was highest for carbapenems (meropenem, 66.4%; imipenem, 62.6%), ciprofloxacin (71.8%), and trimethoprim–sulfamethoxazole (53.1%). Amikacin showed the greatest relative activity and the highest AUC (0.63), but overall discriminatory performance of all tested agents was modest.

**Conclusion:**

MDR *Acinetobacter baumannii* is highly prevalent in intensive care units in this setting and shows extensive resistance to carbapenems, fluoroquinolones, and other commonly used agents. Although amikacin demonstrated comparatively better *in vitro* activity and the highest AUC, its modest discriminatory performance indicates limited reliability as monotherapy. These findings support the use of ICU-specific susceptibility data from endotracheal aspirates to guide empirical therapy and highlight the need for targeted stewardship interventions in high-burden critical care units.

## 1 Introduction

The global rise of antimicrobial resistance (AMR) constitutes one of the most serious threats to modern healthcare, undermining the efficacy of antibiotics and risking a regression to the pre-antibiotic era (World Health Organization, 2023). The problem is particularly critical in tertiary-care hospitals, where Intensive Care Units (ICUs) serve as epicenters for the emergence and dissemination of multidrug-resistant (MDR) pathogens, contributing to higher rates of healthcare-associated infections (HAIs), prolonged hospitalization, and increased mortality (Villarreal-Cruz et al., 2025; Wang et al., 2025). Among Gram-negative pathogens, *A. baumannii* has emerged as a globally recognized opportunistic bacterium due to its extraordinary capacity to acquire resistance determinants and persist in the hospital environment (Howard et al., 2012; Borcan et al., 2025; Foglia et al., 2025). Its carbapenem-resistant strains (CRAB) are listed by both the World Health Organization (WHO) and the Centers for Disease Control and Prevention (CDC) as critical priority pathogens, reflecting their limited therapeutic options and global public health importance (World Health Organization, 2017a,b; Centers for Disease Control and Prevention, 2019).

In neurosurgical and critical care units, the threat posed by MDR *A. baumannii* is particularly severe. Patients frequently undergo invasive procedures such as mechanical ventilation, central venous catheterization, and prolonged antibiotic therapy, which predispose them to Ventilator-Associated Pneumonia (VAP) and bloodstream infections (Shrestha et al., 2019; Teng et al., 2022). Studies have shown that VAP remains one of the most frequent ICU-acquired infections, significantly increasing morbidity, mortality, and healthcare costs (Kalanuria et al., 2014). The risk is even greater in neurosurgical populations, where the combination of neurological injury and extended ventilation duration enhances susceptibility to infection (Sachdeva et al., 2017; Shrestha et al., 2019; Teng et al., 2022; Battaglini et al., 2023). Timely administration of an appropriate antibiotic is critical for favorable patient outcomes. However, extensive use of empirical broad-spectrum antibiotics without regularly updated local susceptibility data continues to drive resistance. A similar pattern is seen in urinary tract infections, where up to half of antibiotic prescriptions are considered inappropriate and many women with uncomplicated UTI receive non-recommended agents and overly long treatment courses (Clark et al., 2021; Kolodziej et al., 2022). This highlights the necessity of localized antimicrobial surveillance and evidence-based stewardship programs tailored to specific hospital units and patient populations.

In addition to institutional determinants, patient demographics, particularly age and sex, can modulate both infection risk and resistance patterns. Studies in urinary tract infections and other severe infections have shown that pathogen distribution and the likelihood of antimicrobial resistance differ substantially across age and sex groups (Magliano et al., 2012; Lo et al., 2013; Waterlow et al., 2024). Understanding such associations in MDR *A. baumannii* infections can guide more individualized treatment decisions.

The present study was designed to investigate the prevalence and antimicrobial resistance patterns of MDR *A. baumannii* isolated from endotracheal aspirates over a 6-year period (2019–2025) in high-dependency and intensive care units of a tertiary-care hospital, with particular attention to their role in ventilator-associated infections. In addition to unit-specific susceptibility profiles, patterns of co-resistance among commonly used antibiotic classes were examined. Receiver operating characteristic (ROC) curve analysis was used to assess the discriminatory performance of key antibiotics against MDR isolates. By integrating long-term local surveillance with formal statistical modeling, this study provides a clinically oriented evidence base to support empirical therapy and to inform antimicrobial stewardship in critical care settings.

## 2 Materials and methods

### 2.1 Materials

Bacteriological media used for the isolation and susceptibility testing of clinical isolates including Blood Agar, MacConkey Agar, and Mueller–Hinton Agar/Broth, were procured from HiMedia Laboratories Pvt. Ltd. (Mumbai, India; bacteriological grade). Bacterial identification and automated MIC determination were performed using the Vitek 2 Compact system with Vitek 2 GN Identification (ID-GN) cards and Vitek 2 Antimicrobial Susceptibility Testing (AST-N406) cards (bioMérieux, Marcy-l'Étoile, France). Antibiotic discs for Kirby–Bauer testing were sourced from HiMedia (India) and included amikacin (30 μg), cefepime (30 μg), gentamicin (10 μg), imipenem (10 μg), meropenem (10 μg), ciprofloxacin (5 μg), trimethoprim–sulfamethoxazole (1.25/23.75 μg), cefoperazone/sulbactam (75/30 μg), and ticarcillin/clavulanic acid (75/10 μg), all meeting CLSI-recommended potency standards. All chemicals used in media preparation and routine laboratory procedures were of analytical grade, and all aqueous solutions were prepared using Milli-Q purified water (Merck Millipore, USA).

### 2.2 Study design, data collection, and microbiological procedures

This was a single-center, retrospective, observational study conducted at JSS Hospital, Mysuru, Karnataka, India, analyzing existing data from the Clinical Microbiology Laboratory database spanning January 2019 until May 2025. The study cohort comprised all non-duplicate clinical bacterial isolates recovered from admitted patients, with demographic data (age, sex) and Antimicrobial Susceptibility Testing (AST) results being extracted. The focus was placed on isolates from critical departments, particularly Neurosurgery, due to the high incidence of mechanical ventilation-associated infections. Inclusion criteria mandated isolates be clinically significant, non-duplicate, and possess complete AST data, while exclusion criteria included surveillance cultures, environmental samples, and samples with colony counts indicative of contamination (e.g., typically < 10^5^ cfu/ml for urine). Bacterial identification was primarily performed using the automated Vitek 2 Compact system and confirmed by standard biochemical tests. During the study period, multiple *Acinetobacter* species were identified, including *A. baumannii*, members of the *A. baumannii* complex, and other *Acinetobacter* spp., reflecting the original laboratory reporting format. Multidrug resistance (MDR) was classified as a resistance phenotype within *A. baumannii* rather than a distinct taxonomic category. However, detailed antimicrobial resistance analysis, demographic stratification, co-resistance network modeling, and ROC curve analysis were performed exclusively on MDR *A. baumannii* isolates recovered from endotracheal (ET) aspirates. AST was formulated using the disc diffusion method (Kirby-Bauer Method) or automated MIC determination via the Vitek 2 Compact system, with results interpreted according to CLSI guidelines applicable for the year of collection. In this study, isolates were classified as multidrug resistant (MDR) if they showed reduced susceptibility to at least one antimicrobial agent in three or more distinct antimicrobial classes, whereas carbapenem-resistant *A. baumannii* (CRAB) was defined as resistant to imipenem and/or meropenem. For the specialized ROC curve analysis against MDR *A. baumannii* isolates from endotracheal (ET) aspirates, susceptibility data for nine specific antibiotics were prioritized: amikacin (30 μg), cefepime (30 μg), gentamicin (10 μg), imipenem (10 μg), meropenem (10 μg), ciprofloxacin (5 μg), trimethoprim-sulfamethoxazole (1.25 μg + 23.75 μg), cefoperazone/sulbactam (75/30 μg), and ticarcillin/clavulanic acid (75/10 μg). This retrospective observational study was approved by the Institutional Ethics Committee of JSS Medical College, Mysuru (Approval No. JSSMC/IEC/070324/04 NCT/2024–25), which included an amendment covering the secondary analysis of anonymized routine clinical microbiology data. The requirement for informed consent was waived due to the retrospective nature of the study.

### 2.3 Statistical analysis

Data were analyzed using SPSS version 22, R version 4.5.1(Web graph), and Python—Jupyter Lite (ROC curve), with statistical significance defined as *p* < 0.05. Descriptive statistics were first used to calculate the frequency and proportion of isolates by age group, sex, and hospital department, thereby establishing the local epidemiological profile. ROC analysis was performed as an exploratory assessment to quantify the degree of separation in minimum inhibitory concentration (MIC) distributions between susceptible and resistant isolates for each antibiotic within this ICU cohort. This approach was intended to evaluate distributional discrimination of MIC values in a high-resistance setting and not to assess antimicrobial efficacy or redefine established susceptibility breakpoints. The area under the curve (AUC) was calculated as a summary measure of discriminatory performance, where an AUC of 1.0 indicates complete separation of MIC distributions and 0.5 reflects no discriminatory ability beyond random classification.

## 3 Results

### 3.1 Demographic distribution of *Acinetobacter baumannii* isolates

The majority of clinical isolates were obtained from patients aged >30 years (51.9%), followed by 13–29 years (29.3%) and 0–12 years (18.7%). Male patients accounted for 74% of the isolates. Department-wise distribution showed that Neurosurgery contributed 55.8% of isolates, followed by General Medicine (20.1%) and Geriatrics (5.3%). Ward-level data indicated that isolates were most frequently recovered from the Neurosurgical ICU (22.1%), general ICU (14.1%), and Surgical ICU (12.9%). MDR *Acinetobacter baumannii* was the most prevalent pathogen (20.2%), followed by *Pseudomonas aeruginosa* (13.6%) and *Klebsiella pneumoniae* ssp. *pneumoniae* (10%) (Supplementary Table 1). Antimicrobial susceptibility testing demonstrated resistance rates of 71.8% for ciprofloxacin, 66.4% for meropenem, and 62.6% for imipenem. Sensitivity rates were 30% for gentamicin and 26.7% for amikacin (Supplementary Table 2). Minimum inhibitory concentration (MIC) analysis showed mean MIC values of 4.8 for imipenem and 5.3 for meropenem. Gentamicin and amikacin demonstrated mean MIC values of 5.9 and 3.6, respectively, while cefoperazone/sulbactam had a mean MIC of 4.2. Highly skewed MIC distributions were observed for ceftizoxime and daptomycin (Supplementary Table 3).

### 3.2 Ward-wise distribution of *A. baumannii* isolates

Ward-wise analysis showed that MDR *A. baumannii* isolates were distributed across multiple hospital units, with the highest proportion observed in the Neurosurgical ICU (22.1%), followed by the Surgical ICU (12.9%), SICU step-down unit (9.3%), and Medical ICU (9.0%). Additional isolates were identified in the RICU and Neuro Surgery Ward, while lower counts were recorded in the CCU, PICU, NICU, and Semi-Private Ward units.

**Figure 1:**
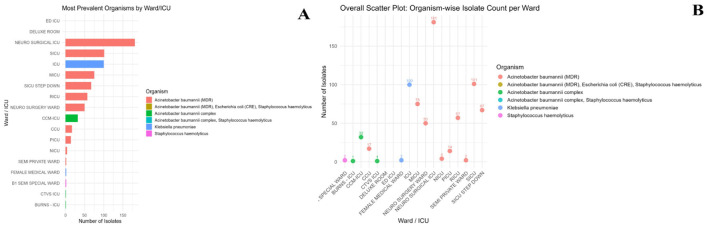
**(A)** Horizontal bar chart depicting the most prevalent clinical bacterial isolates across hospital wards/ICUs, highlighting relative abundance of different organisms. **(B)** Scatter plot illustrating organism-wise distribution patterns and isolate counts across wards/ICUs.

**Figure 2:**
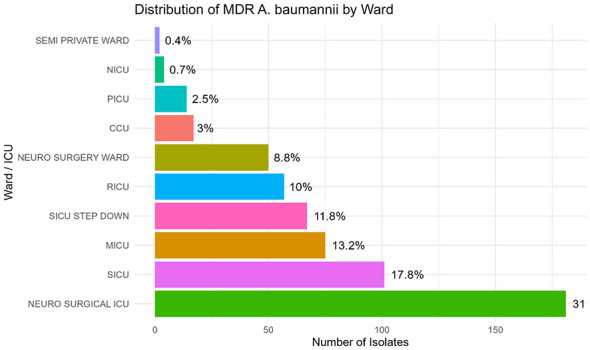
Percentage distribution of MDR *Acinetobacter baumannii* isolates by hospital ward.

Collectively, the intensive care units accounted for more than 67% of the total MDR *A. baumannii* isolates. The organism-wise distribution across wards is presented (Figures 1A, B), and the percentage distribution of MDR *A. baumannii* isolates by hospital ward is shown (Figure 2).

### 3.3 Antibiotic resistance profile of multidrug-resistant *A. baumannii*

**Figure 3:**
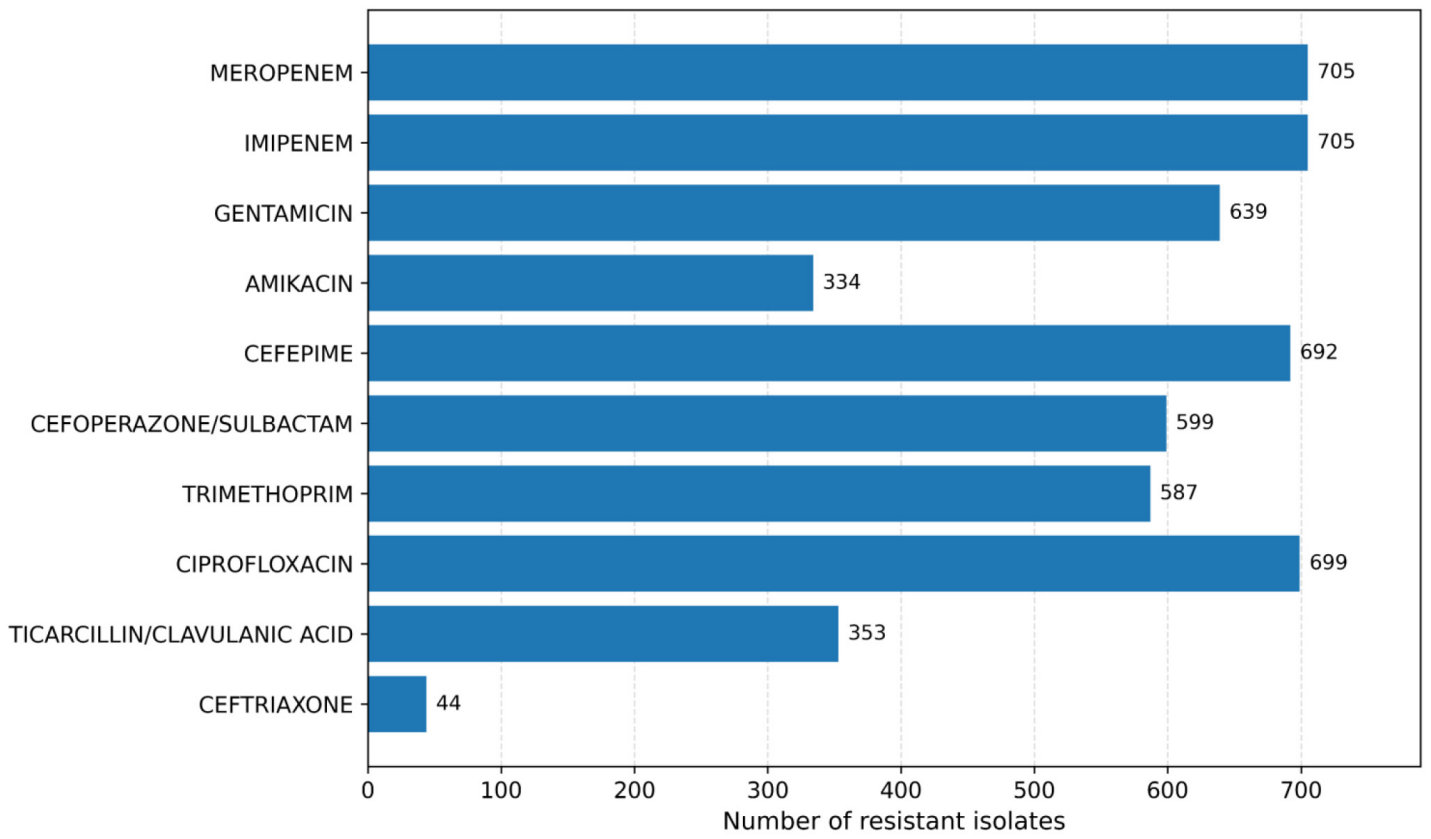
Resistance distribution of MDR *Acinetobacter baumannii* isolates to key antibiotics.

A total of 714 MDR *A. baumannii* isolates were identified from endotracheal aspirate samples. Resistance profiling (Figure 3) demonstrated high resistance counts for meropenem (705 isolates) and imipenem (705 isolates), followed by ciprofloxacin (699 isolates) and cefepime (692 isolates). Gentamicin (639 isolates), cefoperazone/sulbactam (599 isolates), and trimethoprim (587 isolates) also showed substantial resistance. Lower resistance counts were observed for amikacin (334 isolates) and ticarcillin/clavulanic acid (353 isolates). Ceftriaxone exhibited resistance in 44 isolates, representing the lowest resistance count among the antibiotics tested.

**Figure 4:**
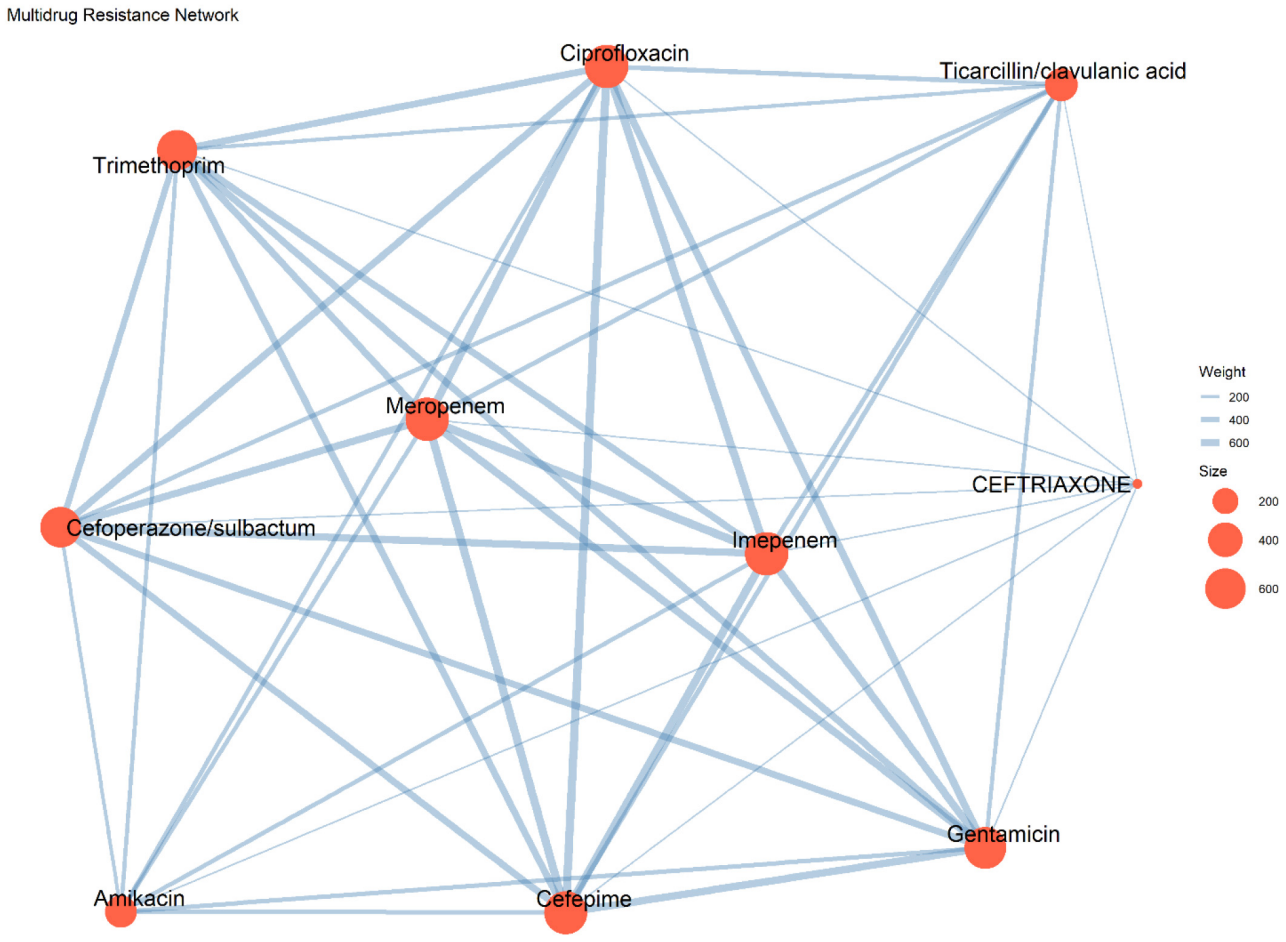
Multidrug resistance network of *Acinetobacter baumannii*. Nodes represent antibiotics, with node size proportional to the number of resistant isolates and edge thickness reflecting the strength of co-resistance between agents.

The MDR network of *A. baumannii* is presented (Figure 4). In the network, nodes represent antibiotics and node size corresponds to the number of resistant isolates, while connecting lines indicate co-resistance between antibiotic pairs. The thickness of the connecting lines reflects the strength of co-resistance associations. Carbapenems (imipenem, meropenem), cephalosporins (ceftriaxone, cefepime), aminoglycosides (amikacin, gentamicin), ciprofloxacin, and β-lactam/β-lactamase inhibitor combinations (cefoperazone/sulbactam, ticarcillin/clavulanic acid) formed major interconnected groups within the network.

### 3.4 Multidrug resistance *A. baumannii* antibiotic resistance by patient age

**Figure 5:**
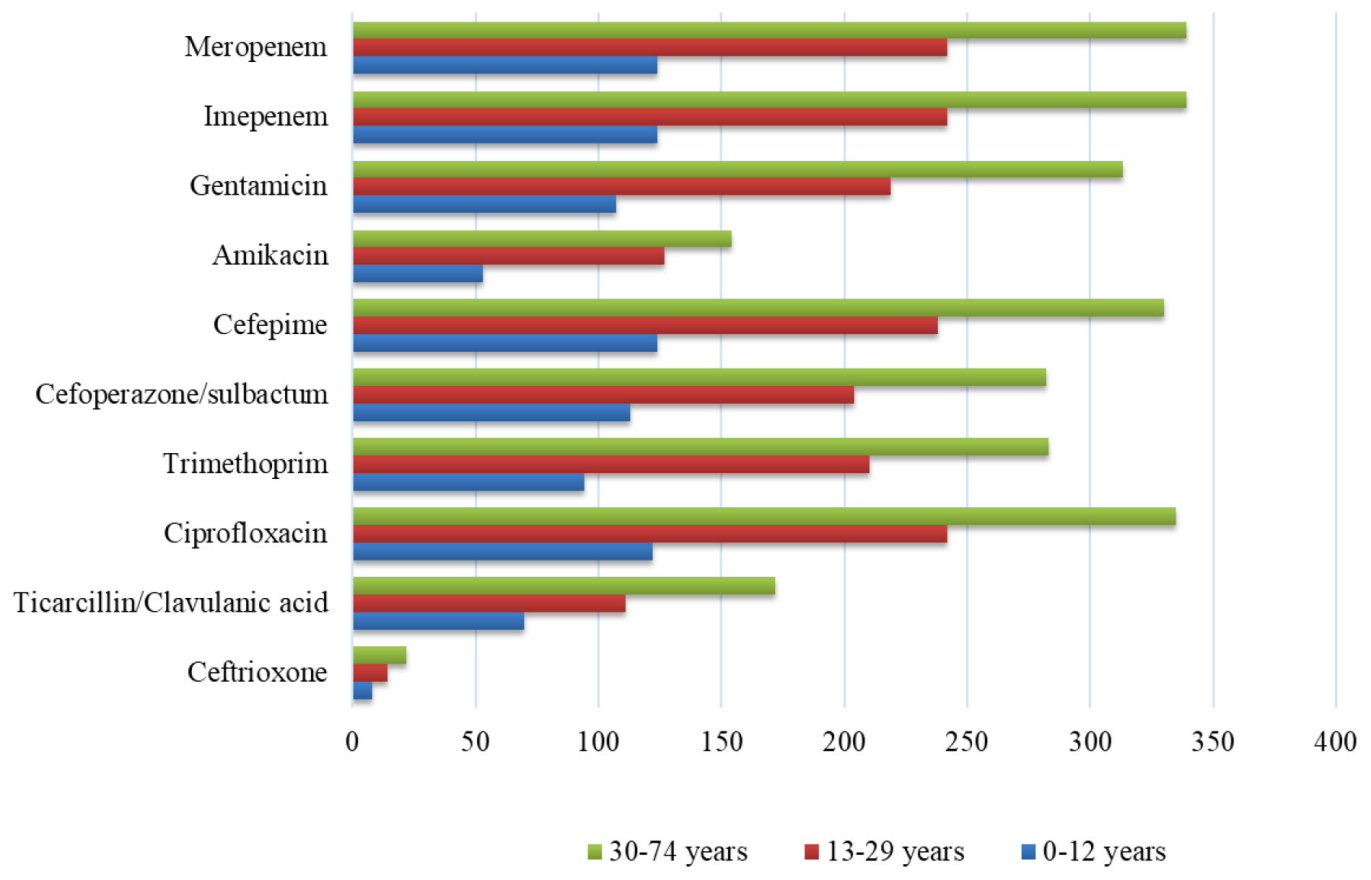
Antibiotic Resistance Patterns of Multidrug-Resistant Acinetobacter baumannii Stratified by Patient Age. This chart compares the number of resistant isolates for ten antibiotics across three age groups (0–12 years, 13–29 years, and 30–74 years). The 30–74 years age group consistently shows the highest number of resistant isolates for almost every antibiotic tested.

The bar chart (Figure 5) illustrates the distribution of antibiotic resistance in *A. baumannii* isolates across three distinct patient age groups: 0–12 years, 13–29 years, and 30–74 years. The data shows that the 30–74 years age group consistently exhibits the highest number of resistant isolates for virtually all ten antibiotics tested, including critical drugs like meropenem, cefepime, and ciprofloxacin. Conversely, the 0–12 years group generally shows the lowest resistance counts (Supplementary Table 4). This pattern indicates that resistance to *A. baumannii* is most prevalent in older adult and middle-aged patients, which has significant implications for clinical management and infection control in these populations. Values are presented as number of resistant isolates with percentages in brackets.

### 3.5 Sex-wise distribution of antimicrobial resistance in MDR *A. baumannii*

The sex-wise distribution of antimicrobial resistance among MDR *A. baumannii* isolates is presented (Supplementary Table 5). Among male patients, resistance was most frequently observed for ciprofloxacin (508 isolates), cefepime (501 isolates), imipenem (512 isolates), meropenem (511 isolates), gentamicin (460 isolates), cefoperazone/sulbactam (431 isolates), and trimethoprim (424 isolates). Resistance to ticarcillin/clavulanic acid (256 isolates) and amikacin (245 isolates) was also recorded.

**Figure 6:**
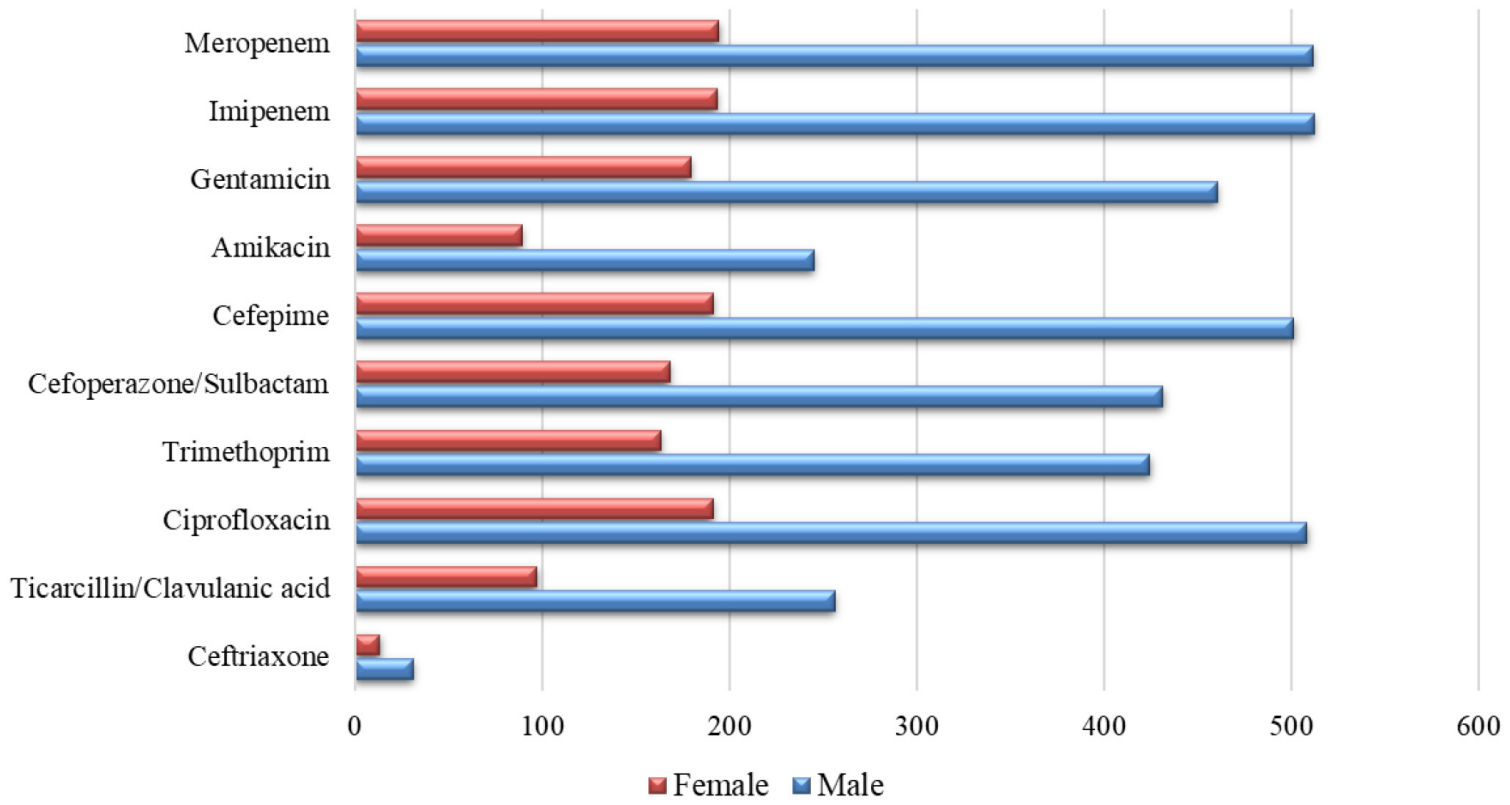
Multidrug-resistant *Acinetobacter baumannii* antibiotic resistance by patient sex. This bar chart compares the number of resistant isolates for ten antibiotics between female and male patients. The male patient group consistently shows a higher number of resistant isolates for all tested antibiotics.

Among female patients, resistance was observed for ciprofloxacin (191 isolates), cefepime (191 isolates), imipenem (193 isolates), meropenem (194 isolates), gentamicin (179 isolates), cefoperazone/sulbactam (168 isolates), and trimethoprim (163 isolates). Amikacin resistance was identified in 89 isolates. Male patients had a consistently higher number of multidrug-resistant *A. baumannii* isolates than female patients for all ten antibiotics analyzed (Figure 6). This imbalance may reflect a higher burden of comorbidities, more frequent exposure to invasive procedures, or longer ICU stays among male patients in this cohort. Although the present study was not designed to determine causal mechanisms, the clear predominance of resistant isolates in men suggests that sex-related clinical factors may contribute to the selection and persistence of MDR strains. This pattern should be taken into account when planning infection-prevention strategies and when developing empirical treatment protocols for ventilated patients at risk of MDR *A. baumannii* infection.

### 3.6 Sensitivity analysis

**Figure 7:**
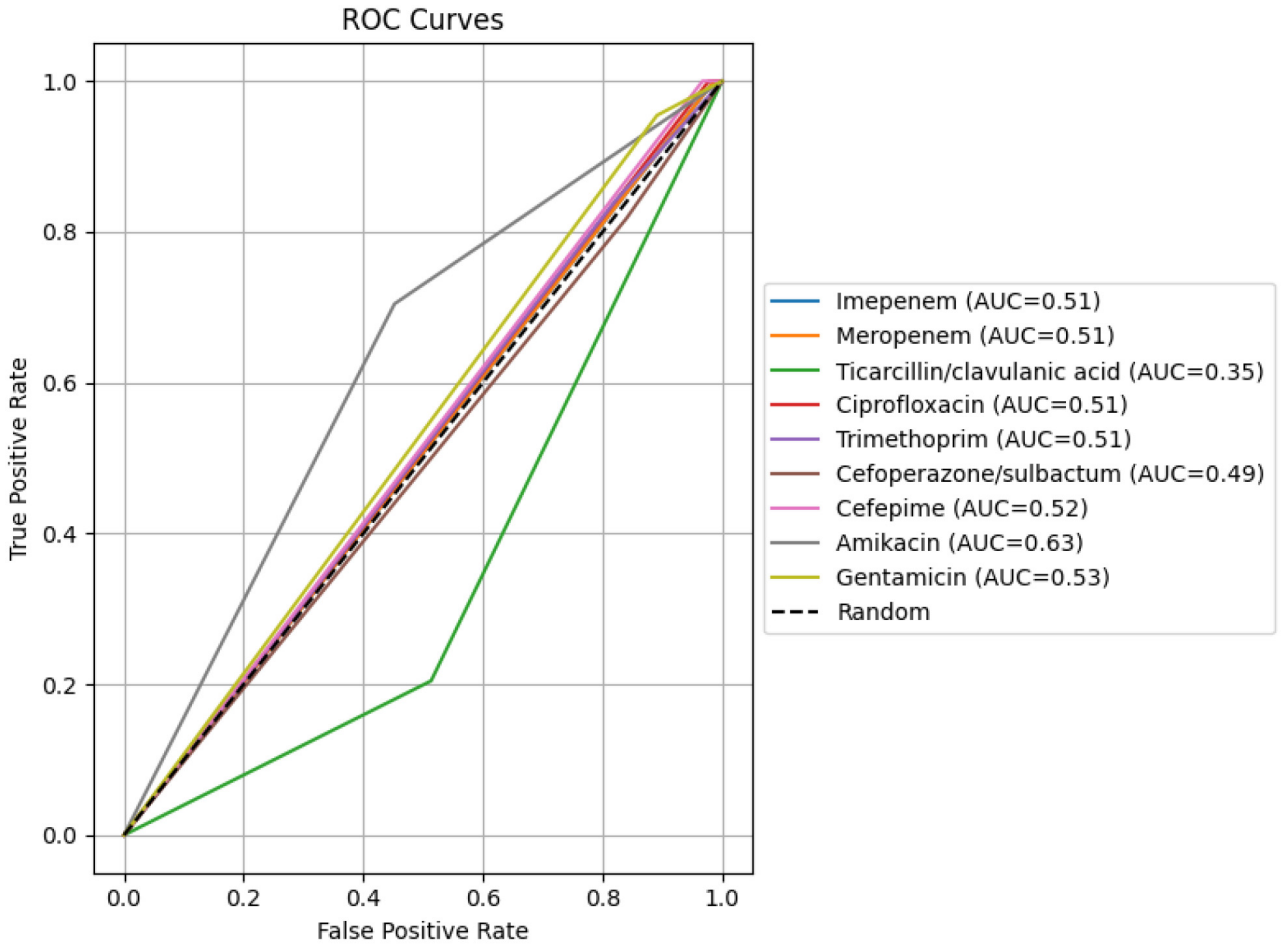
Receiver operating characteristic (ROC) curves for the efficacy of nine antibiotics against multidrug-resistant *Acinetobacter baumannii* isolates obtained from endotracheal (ET) aspirates.

The ROC analysis demonstrated that most antibiotics exhibited AUC values close to 0.50, indicating limited discriminatory ability of MIC values in distinguishing resistant from susceptible isolates (Figure 7). Amikacin showed modest discrimination (AUC = 0.63), while ticarcillin/clavulanic acid demonstrated poor discrimination (AUC = 0.35). These findings suggest substantial overlap in MIC distributions between susceptibility categories in this cohort.

## 4 Discussion

Multidrug-resistant *Acinetobacter baumannii* emerged in this analysis as the predominant nosocomial pathogen in a tertiary-care setting, accounting for a substantial proportion of clinical isolates and surpassing other Gram-negative organisms. The consistently high number of MDR *A. baumannii* cases over 6 years suggests that it has become a persistent problem in high-risk hospital units. Most isolates were recovered from middle-aged and older adults, with a marked male predominance, and were predominantly derived from high-dependency and intensive care units, particularly the Neurosurgical ICU. This distribution is consistent with the high burden of invasive procedures, prolonged ventilation and intensive monitoring in these units, which are well-recognized drivers of MDR *A. baumannii* transmission in contemporary ICU cohorts (Silva et al., 2024; Zingg et al., 2024). Within this study population, imipenem- and meropenem-resistant isolates were highly prevalent, which translated into markedly constrained therapeutic choices. Similar patterns of carbapenem resistance in *A. baumannii* have been reported from recent ICU-based studies, including those conducted during and after the COVID-19 pandemic (Silva et al., 2024; Zingg et al., 2024).

The high prevalence of MDR *A. baumannii* in intensive care units at this institution aligns with other investigations that have highlighted the combined impact of extensive antimicrobial exposure, invasive devices, and the organism's ability to persist in the hospital environment (Zingg et al., 2024). However, the magnitude and persistence of resistance observed in our dataset reinforce the need for continuous local surveillance rather than reliance on regional or national resistance estimates alone. During the COVID-19 pandemic, increased ICU occupancy, prolonged ventilatory support, and heavy use of broad-spectrum antibiotics further intensified conditions favoring the emergence and spread of resistant and biofilm-forming *A. baumannii* strains (Silva et al., 2024; Chen et al., 2025). The carbapenem resistance levels observed here are in agreement with a recent systematic review reporting resistance exceeding 80% in many Asian countries (Zhang et al., 2024). Indian data similarly document widespread resistance to imipenem, meropenem and other commonly used agents among ventilator-associated isolates from tertiary-care centers (Goenka et al., 2025). In our cohort, the high frequency of carbapenem resistance effectively limited first-line therapeutic options, reflecting the practical clinical challenge faced in this ICU setting. At the molecular level, extensive carriage and co-occurrence of carbapenemase genes, particularly *blaOXA-23, blaNDM-1* and *blaIMP-1* have been described in *A. baumannii* isolates from India, illustrating the genetic complexity underpinning carbapenem resistance in this region (Saranathan et al., 2015; Kumar et al., 2019; Datta et al., 2025).

The multidrug resistance pattern observed in this study, including frequent resistance to cephalosporins, aminoglycosides and β-lactam/β-lactamase inhibitor combinations, is compatible with the coexistence of multiple resistance determinants. Previous work has shown that MDR *A. baumannii* often harbors combinations of class A, B, and D β-lactamases together with virulence traits such as biofilm formation, which enhance environmental persistence and complicate therapeutic management (Gautam et al., 2023; Arif et al., 2024). In this study, an MDR co-resistance network was constructed to better understand the relationships between resistance traits within the ICU cohort (Figure 4). The analysis revealed that ceftriaxone, meropenem, and ciprofloxacin functioned as central nodes with multiple connections to other antibiotics, indicating frequent co-occurrence of resistance across several antimicrobial classes within the same isolates. This clustering pattern suggests that resistance traits in this setting are structurally interconnected rather than occurring independently. These findings provide additional insight into the resistance architecture of MDR *A. baumannii* in this institution and highlight the potential for co-selection of multidrug resistance during empirical use of broad-spectrum antibiotics.

Age-stratified analyses confirmed that resistance was most pronounced among older adults, whereas pediatric patients contributed relatively few MDR *A. baumannii* isolates. In this study, the higher resistance observed among older patients likely reflects greater comorbidity burden, prolonged hospitalization, and cumulative prior antibiotic exposure within this ICU cohort. Higher resistance in older age groups is consistent with previously reported risk factors such as a greater comorbidity burden, cumulative prior antibiotic exposure and longer hospital stays. By contrast, sex did not significantly influence resistance patterns, suggesting that clinical factors such as severity of illness and device use may have a greater impact than demographic sex differences in this setting. Environmental transmission remains an important factor in the persistence of *A. baumannii* in healthcare facilities. The predominance of isolates from ventilated and high-dependency units in our dataset further supports the role of device-associated transmission in sustaining MDR *A. baumannii*. ICU outbreak investigations have demonstrated that this organism can survive on bed rails, ventilator surfaces, monitors, suction ports, and other high-touch items, thereby facilitating rapid spread between critically ill patients (Zingg et al., 2024). The isolation of biofilm-forming strains during COVID-19 surges further underscores the capacity of *A. baumannii* to persist in humid ICU environments and to withstand routine cleaning and disinfection procedures (Silva et al., 2024; Chen et al., 2025).

Receiver operating characteristic analysis provided additional insight into the performance of individual antibiotics against MDR *A. baumannii* from endotracheal aspirates. Amikacin achieved the highest area under the curve, indicating better discriminatory ability than the other agents evaluated, but its AUC still reflected only moderate separation between susceptible and resistant isolates. Gentamicin and cefepime showed AUC values only slightly above 0.5, while imipenem, meropenem, ciprofloxacin, trimethoprim and cefoperazone/sulbactam demonstrated values close to random classification. Ticarcillin/clavulanic acid yielded an AUC clearly below 0.5, confirming its lack of utility for guiding therapy in this context. Overall, these findings indicate that no single antibiotic reliably predicts susceptibility in MDR *A. baumannii*, which is consistent with current evidence that heterogeneous resistance mechanisms in this pathogen limit the predictive value of phenotypic susceptibility patterns alone and that empirical treatment frequently relies on combination regimens (Kumar et al., 2021). These findings represent statistical separation of MIC distributions and should not be interpreted as direct measures of antibiotic efficacy.

From a clinical and antimicrobial stewardship perspective, the high rate of carbapenem resistance, extensive co-resistance across multiple drug classes, and only modest ROC performance observed in this study have important implications for empirical therapy in ventilated patients. Carbapenems and other broad-spectrum agents cannot be regarded as predictably effective options against MDR *A. baumannii* and should be prescribed in alignment with up-to-date local susceptibility data and unit-specific guidelines. The comparatively better activity of amikacin and the retained efficacy of colistin suggest that these agents remain key components of treatment strategies; however, their use is constrained by toxicity and the risk of further resistance selection. Taken together, the resistance architecture identified in this study underscores the limited reliability of single-agent empirical therapy in high-risk ventilated patients. These observations support the need for strengthened antimicrobial stewardship programmes and strict infection-prevention measures in high-risk units, as well as for integration of molecular tools to support outbreak investigation, resistance-gene surveillance and refinement of antibiotic policy (Zhang et al., 2024; Karampatakis et al., 2025). Future research using whole-genome sequencing and longitudinal surveillance could provide deeper insight into clonal spread, resistance evolution, and the impact of interventions in high-risk units. Importantly, this study provides long-term ICU-specific epidemiological data integrating resistance profiling, co-resistance network modeling, and ROC-based discrimination analysis within a single institutional cohort.

This study has several notable strengths. A long observation period from 2019 to 2025 was covered, a large number of non-duplicate clinical isolates were analyzed, and attention was focused specifically on MDR *A. baumannii* from endotracheal aspirates in neurosurgical and intensive care units, directly reflecting the population at risk for ventilator-associated infections. Comprehensive antimicrobial susceptibility testing was combined with network-based assessment of co-resistance and ROC analysis, providing a multifaceted view of resistance epidemiology in this setting. Certain limitations should also be acknowledged. The investigation was conducted at a single tertiary-care hospital and did not incorporate molecular typing, thereby precluding detailed assessment of clonal spread and specific resistance mechanisms. Although carbapenem resistance was confirmed phenotypically using the Vitek 2 system and the Kirby–Bauer disk diffusion method in accordance with CLSI guidelines, molecular characterization of specific resistance determinants (e.g., OXA-type carbapenemases, NDM, or ISAba1-associated genes) was not performed; therefore, the underlying genetic mechanisms contributing to carbapenem resistance in these isolates could not be determined. As this was a single-center study, the findings cannot be generalized to other healthcare settings beyond similar ICU environments. Furthermore, several important clinical variables were not available in this retrospective dataset, including differentiation between colonization and true infection (particularly ventilator-associated pneumonia), prior antimicrobial exposure, and patient outcomes such as mortality or length of ICU stay. While resistance patterns were characterized at the microbiological level, associations with clinical outcomes could not be assessed. Nevertheless, the extended study period and inclusion of high-risk critical care units provide meaningful local epidemiological data to support empirical antibiotic selection and infection-control strategies targeting MDR *A. baumannii* in intensive care settings.

## Conclusion

This retrospective study demonstrates a high burden of MDR *A. baumannii* in intensive care units, particularly among mechanically ventilated patients. MDR *A. baumannii* constituted a substantial proportion of isolates and exhibited extensive resistance to carbapenems, fluoroquinolones, and several cephalosporins, indicating limited effectiveness of commonly used empirical therapies. Although amikacin showed comparatively better *in vitro* activity and the highest area under the ROC curve, its modest discriminatory performance highlights the lack of reliably effective single-agent options. Resistance was more frequent among older adults, while no consistent association with sex was observed. The marked concentration of MDR *A. baumannii* in endotracheal aspirates from neurosurgical and other critical care units, together with uniformly high carbapenem resistance and limited predictive performance of commonly used antibiotics, supports the use of ICU-specific antibiograms and targeted stewardship interventions rather than routine reliance on broad empirical regimens. These findings are consistent with global trends and emphasize the importance of unit-level strategies to mitigate antimicrobial resistance in critical care settings.

## Data Availability

The original contributions presented in the study are included in the article/Supplementary material, further inquiries can be directed to the corresponding author/s.
