# Supplementary material for: Prevalence and antimicrobial resistance of Acinetobacter baumannii isolated from endotracheal aspirates in a tertiary-care intensive care unit from South India
Source: Front Microbiol. 2026 Apr 15;17:1784001. doi: 10.3389/fmicb.2026.1784001 (PMC13125122; doi:10.3389/fmicb.2026.1784001)
Supplement: Supplementary file 1 [file Table_1.docx]

**Supplementary Material**

**Supplementary Table 1.** Demographic characteristics and distribution of clinical bacterial isolates by age group, sex, hospital department, and ward during the study period (2019–2025). N, number of isolates; %, percentage of all isolates (n = 3540).

| Characteristics | | **N** | **%** |
| --- | --- | --- | --- |
| **Age** | 0-12 years | 664 | 18.7 |
|  | 13-29 years | 1038 | 29.3 |
|  | 30 years and above | 1838 | 51.9 |
| **Sex** | Male | 2620 | 74.0 |
|  | Female | 920 | 26.0 |
| **Department** | Cardiology | 36 | 1.0 |
|  | Cardiothoracic | 8 | 0.2 |
|  | Critical Care Medicine | 92 | 2.6 |
|  | Dental | 1 | 0.0 |
|  | Emergency Medicine | 15 | 0.4 |
|  | ENT | 26 | 0.7 |
|  | Gastroenterology | 9 | 0.3 |
|  | Geriatric | 188 | 5.3 |
|  | Interventional Neuro Radiology | 8 | 0.2 |
|  | Medical Oncology | 7 | 0.2 |
|  | Medicine | 710 | 20.1 |
|  | Nephrology | 84 | 2.4 |
|  | Neurology | 83 | 2.3 |
|  | Neurosurgery | 1974 | 55.8 |
|  | OBG | 9 | 0.3 |
|  | Orthopedic | 16 | 0.5 |
|  | Pediatric Surgery | 4 | 0.1 |
|  | Paediatrics | 67 | 1.9 |
|  | Plastic Surgery | 16 | 0.5 |
|  | Respiratory Medicine | 84 | 2.4 |
|  | Rheumatology | 8 | 0.2 |
|  | Surgery | 72 | 2.0 |
|  | Surgical Gastroenterology | 11 | 0.3 |
|  | Surgical Oncology | 5 | 0.1 |
|  | Urology | 6 | 0.1 |
|  | Vascular Surgery | 1 | 0.0 |
| **Ward** | B1 semi special ward | 6 | 0.2 |
|  | Burns – ICU | 3 | 0.1 |
|  | CCM-ICU | 99 | 2.8 |
|  | CCU | 88 | 2.5 |
|  | Covid-hdu ward (mentw) | 1 | 0.0 |
|  | Covid-isol ICU without ventilator (emg) | 1 | 0.0 |
|  | CTVS ICU | 2 | 0.1 |
|  | Ctvs icu step down | 7 | 0.2 |
|  | Deluxe room | 5 | 0.1 |
|  | EDICU | 13 | 0.4 |
|  | Emd green zone | 1 | 0.0 |
|  | Female medical ward | 3 | 0.1 |
|  | Female neurology ward | 1 | 0.0 |
|  | Female urology ward | 4 | 0.1 |
|  | Geriatrics ward | 4 | 0.1 |
|  | ICU | 500 | 14.1 |
|  | ICU step down | 4 | 0.1 |
|  | Isolation ICU | 34 | 1.0 |
|  | Male ent ward | 14 | 0.4 |
|  | Male medical ward | 2 | 0.1 |
|  | Male neurology ward | 2 | 0.1 |
|  | Male respiratory medicine | 1 | 0.0 |
|  | Male urology ward | 9 | 0.3 |
|  | MICU | 317 | 9.0 |
|  | Neuro surgery ward | 337 | 9.5 |
|  | Neuro surgical ICU | 783 | 22.1 |
|  | NICU | 17 | 0.5 |
|  | Paediatrics ward | 3 | 0.1 |
|  | PICU | 77 | 2.2 |
|  | Private ward 3 sharing-female | 22 | 0.6 |
|  | Private ward 3 sharing-male | 86 | 2.4 |
|  | Private ward AC | 18 | 0.5 |
|  | Private ward non AC | 50 | 1.4 |
|  | RICU | 236 | 6.7 |
|  | Semi private ward | 6 | 0.2 |
|  | SICU | 455 | 12.9 |
|  | SICU step down | 329 | 9.3 |
| **Isolates** | *Acinetobacter baumannii* | 329 | 9.3 |
|  | *Acinetobacter baumannii* (MDR) | 714 | 20.2 |
|  | *Acinetobacter baumannii complex* | 428 | 12.1 |
|  | *Acinetobacter junii* | 3 | 0.1 |
|  | *Acinetobacter lwoffii* | 12 | 0.3 |
|  | *Acinetobacter species* | 5 | 0.1 |
|  | *Aeromonas salmonicida* | 1 | 0.0 |
|  | *Alpha Haemolytic Streptococci* | 19 | 0.5 |
|  | *BETA HAEMOLYTIC STREPTOCOCCI* | 2 | 0.1 |
|  | *Brevundimonas diminuta / vesicularis* | 2 | 0.1 |
|  | *Burkholderia cepacia* | 7 | 0.2 |
|  | *Candida albicans* | 35 | 1.0 |
|  | *Candida ciferri grown in culture* | 1 | 0.0 |
|  | *Candida glabrata* | 3 | 0.1 |
|  | *Candida guilliermondii* | 5 | 0.1 |
|  | *Candida krusei* | 1 | 0.0 |
|  | *Candida lusitaniae* | 1 | 0.0 |
|  | *Candida parapsilosis* | 1 | 0.0 |
|  | *Candida tropicalis* | 44 | 1.2 |
|  | *Chryseobacterium gleum* | 1 | 0.0 |
|  | *Chryseobacterium indologenes* | 3 | 0.1 |
|  | *Citrobacter freundii* | 3 | 0.1 |
|  | *Citrobacter koseri* | 3 | 0.1 |
|  | *Citrobacter species* | 3 | 0.1 |
|  | *Coagulase negative staphylococcus* | 1 | 0.0 |
|  | *Elizabethkingia meningoseptica* | 2 | 0.1 |
|  | *Enterobacter aerogenes* | 11 | 0.3 |
|  | *Enterobacter cloacae complex* | 27 | 0.8 |
|  | *Enterobacter cloacae ssp cloacae* | 23 | 0.6 |
|  | *Enterobacter species* | 1 | 0.0 |
|  | *Enterococci.* | 11 | 0.3 |
|  | *Enterococcus faecium* | 2 | 0.1 |
|  | *Escherichia coli* | 90 | 2.5 |
|  | *Escherichia coli (CRE)* | 20 | 0.6 |
|  | *Group A streptococci* | 1 | 0.0 |
|  | *Haemophilus influenzae* | 7 | 0.2 |
|  | *Hafnia alvei* | 1 | 0.0 |
|  | *Klebsiella neumonia ssp neumonia (MDRO)* | 271 | 7.7 |
|  | *Klebsiella pneumoniae* | 91 | 2.6 |
|  | *Klebsiella neumonia (MDR)* | 155 | 4.4 |
|  | *Klebsiella neumonia ssp ozaenae* | 3 | 0.1 |
|  | *Klebsiella neumonia ssp pneumoniae* | 353 | 10.0 |
|  | *Morganella morganii ssp morganii* | 3 | 0.1 |
|  | *Non haemolytic streptococci* | 3 | 0.1 |
|  | *Pneumococci* | 4 | 0.1 |
|  | *Proteus mirabilis* | 11 | 0.3 |
|  | *Proteus vulgaris* | 1 | 0.0 |
|  | *Providencia rettgeri* | 2 | 0.1 |
|  | *Providencia stuartii* | 1 | 0.0 |
|  | *Pseudomonas aeruginosa* | 483 | 13.6 |
|  | *Pseudomonas aeruginosa (MDR)* | 96 | 2.7 |
|  | *Pseudomonas fluorescens* | 5 | 0.1 |
|  | *Pseudomonas luteola* | 4 | 0.1 |
|  | *Pseudomonas putida* | 3 | 0.1 |
|  | *Serratia fonticola* | 1 | 0.0 |
|  | *Serratia marcescens* | 42 | 1.2 |
|  | *Sphingomonas paucimobilis* | 7 | 0.2 |
|  | *Staphylococcus aureus* | 41 | 1.2 |
|  | *Staphylococcus aureus (MRSA)* | 36 | 1.0 |
|  | *Staphylococcus epidermidis* | 2 | 0.1 |
|  | *Staphylococcus haemolyticus* | 11 | 0.3 |
|  | *Staphylococcus hominis ssp hominis* | 2 | 0.1 |
|  | *Stenotrophomonas maltophilia* | 68 | 1.9 |
|  | *Streptococcus pneumoniae* | 8 | 0.2 |
|  | *Streptococcus species* | 10 | 0.3 |

*MDR classification refers to resistance phenotype within Acinetobacter baumannii isolates as defined in Section 2.2 and does not represent a separate taxonomic category. Species identification categories reflect the terminology used in the hospital laboratory reporting system.*

**Supplementary Table 2.** Antimicrobial susceptibility profile of clinical isolates showing the frequency of isolates categorized as susceptible (S), intermediate (I), resistant (R), or not applicable (NA).

| **Antibiotics** | **I** | **S** | **R** | **NA** |
| --- | --- | --- | --- | --- |
| Ceftriaxone | 7(0.2) | 179(5.1) | 926(26.2) | 2428(68.6) |
| Imipenem | 174(4.9) | 737(20.8) | 2216(62.6) | 413(11.7) |
| Meropenem | 42(1.2) | 763(21.6) | 2349(66.4) | 386(10.9) |
| Ticarcillin/Clavulanic acid | 75(2.1) | (4.1) | 778(22.0) | 2543(71.8) |
| Micafungin | - | 80(2.3) | 2(0.1) | 3458(97.7) |
| Nalidixic acid | - | 85(2.4) | 332(9.4) | 3123(88.2) |
| Voriconazole | - | 86(2.4) | 4(0.1) | 3450(97.5) |
| Ceftizoxime | - | - | 6(0.2) | 3534(99.8) |
| Ciprofloxacin | 84(2.4) | 622(17.6) | 2541(71.8) | 293(8.3) |
| Trimethoprim/Sulfamethoxazole | - | 775(21.9) | 1881(53.1) | 884(25.0) |
| Daptomycin | - | 81(2.3) | - | 3459(97.7) |
| Cefuroxime | 4(0.1) | 86(2.4) | 858(24.2) | 2592(73.2) |
| Fluconazole | - | 83(2.3) | 3(0.1) | 3454(97.6) |
| Penicillin -G | 1(0.0) | 34(1.0) | 17(0.5) | 3488(98.5) |
| Cefoperazone/sulbactam | 271(7.7) | 815(23.0) | 1992(56.3) | 462(13.1) |
| Tetracycline | 6(0.2) | 136(3.8) | 36(1.0) | 3362(95.0) |
| Cefixime | - | 7(0.2) | 6(0.2) | 3527(99.6) |
| Gentamicin (high-level) | - | 5(0.1) | 6(0.2) | 3529(99.7) |
| Aztreonam | 34(1.0) | 131(3.7) | 210(5.9) | 3165(89.4) |
| Cefpodoxime | - | 7(0.2) | 1(0.0) | 3532(99.8) |
| Netilmicin | 1(0.0) | 5(0.1) | 12(0.3) | 3522(99.5) |
| Cefepime | 121(3.4) | 700(19.8) | 2297(64.9) | 422(11.9) |
| Ertapenem | 15(0.4) | 267(7.5) | 531(15.0) | 2727(77.0) |
| Oxacillin | - | 40(1.1) | 54(1.5) | 3446(97.3) |
| Ceftazidime/Avibactam | - | 7(0.2) | 13(0.4) | 3520(99.4) |
| Ethambutol | - | 2(0.1) | 2(0.1) | 3520(99.4) |
| Piperacillin | 1(0.0) | - | 1(0.0) | 3538(99.9) |
| Amikacin | 221(6.2) | 945(26.7) | 1317(37.2) | 1057(29.9) |
| Cefaclor | 1(0.0) | 2(0.1) | 8(0.2) | 3529(99.7) |
| Flucytosine | 3(0.1) | 60(1.7) | 5(0.1) | 3472(98.1) |
| Tobramycin | - | 5(0.1) | 13(0.4) | 3522(99.5) |
| Cefazolin | - | 1(0.0) | - | 3522(99.5) |
| Gentamicin | 110(3.1) | 1061(30.0) | 1940(54.8) | 429(12.1) |

* *Gentamicin high-level testing was performed only for Enterococcus spp. according to CLSI guidelines*

**Supplementary Table 3.** Descriptive statistics of patient age and antimicrobial susceptibility test values, including mean, median, mode, standard deviation, skewness, and kurtosis.

| **Antibiotic** | **Mean** | **Median** | **Mode** | **Std. Deviation** | **Skewness** | **Kurtosis** |
| --- | --- | --- | --- | --- | --- | --- |
| CEFTRIAXONE | 2.14 | 0.00 | 0.00 | 3.46 | 1.02 | -0.89 |
| IMIPENEM | 4.80 | 7.0 | 7.0 | 2.7984 | -0.764 | -1.130 |
| MEROPENEM | 5.332 | 7.0 | 7.0 | 2.6442 | -1.103 | -0.213 |
| TICARCILLIN/  CLAVULANIC ACID | 1.445 | 0.00 | 0.0 | 2.49 | 1.22 | -0.43 |
| MICAFUNGIN | 0.5 | 0.00 | 0.00 | 0.57 | 6.61 | 42.03 |
| NALIDIXIC ACID | 0.52 | 0.000 | 0.00 | 1.49 | 2.54 | 4.06 |
| VORICONAZOLE | 0.71 | 0.007 | 0.00 | 0.00 | 6.28 | 37.74 |
| CEFTIZOXIME | 0.001 | 0.000 | 0.0 | 0.037 | 26.5 | 703.99 |
| CIPROFLOXACIN | 6.02 | 8.00 | 8.0 | 3.05 | -1.01 | -0.59 |
| TRIMETHOPRIM/  SULFAMETHOXAZOLE | 6.48 | 7.00 | 10.0 | 4.17 | -0.68 | -1.24 |
| DAPTOMYCIN | 0.02 | 0.00 | 0.0 | 0.19 | 8.61 | 88.48 |
| CEFUROXIME | 1.69 | 0.00 | 0.0 | 2.9 | 1.21 | -0.50 |
| FLUCONAZOLE | 0.10 | 0.00 | 0.0 | 0.74 | 7.64 | 57.4 |
| CEFOPERAZONE/  SULBACTAM | 4.20 | 6.00 | 6.0 | 2.29 | -0.83 | -0.90 |
| TETRACYCLINE | 0.10 | 0.000 | 0.0 | 0.63 | 6.23 | 37.4 |
| AZTREONAM | 0.48 | 0.00 | 0.0 | 2.64 | 1.4 | 0.2 |
| OXACILLIN | 0.05 | 0.00 | 0.0 | 0.36 | 7.0 | 49.8 |
| CEFTAZIDIME/AVIBACTAM | 0.005 | 0.00 | 0.0 | 0.93 | 20.27 | 419.27 |
| ETHAMBUTOL | 0.00 | 0.00 | 0.0 | 0.01 | 59.4 | 3540.00 |
| AMIKACIN | 3.64 | 4.00 | 0.0 | 3.01 | -0.14 | -173 |
| FLUCYTOSINE | 0.70 | 0.00 | 0.0 | 0.52 | 7.49 | 54.91 |
| TOBRAMYCIN | 0.006 | 0.00 | 0.0 | 0.10 | 18.24 | 337.65 |
| GENTAMICIN | 5.88 | 8.00 | 8.0 | 2.92 | -1.18 | -0.11 |

**Supplementary Table 4.** Age-wise distribution of antimicrobial resistance to individual antibiotics among multidrug-resistant *Acinetobacter baumannii* isolates. Data are presented as the number of resistant isolates, with the percentage of MDR *A. baumannii* isolates within each age group shown in parentheses.

| **Antibiotic** | **Age** | | |
| --- | --- | --- | --- |
|  | 0-12 years | 13-29 years | 30-74 years |
| Ceftrioxone | 8(6.4) | 14(5.7) | 22(6.4) |
| Ticarcillin/  Clavulanic acid | 70(56.0) | 111(44.9) | 172(50.3) |
| Ciprofloxacin | 122(97.6) | 242(98.0) | 335(98.0) |
| Trimethoprim | 94(75.2) | 210(85.0) | 283(82.7) |
| Cefoperazone/  sulbactam | 113(90.4) | 204(82.6) | 282(82.5) |
| Cefepime | 124(99.2) | 238(96.4) | 330(96.5) |
| Amikacin | 53(42.4) | 127(51.4) | 154(45.0) |
| Gentamicin | 107(85.6) | 219(88.7) | 313(91.5) |
| Imipenem | 124(99.2) | 242(98.0) | 339(99.1) |
| Meropenem | 124(99.2) | 242(98.0) | 339(99.1) |

**Supplementary Table 5.** Sex-wise distribution of antimicrobial resistance to individual antibiotics among multidrug-resistant *Acinetobacter baumannii* isolates. Data are presented as the number of resistant isolates, with the percentage of MDR *A. baumannii* isolates within each sex group shown in parentheses.

| **Antibiotic** | **Sex** | |
| --- | --- | --- |
|  | **Male** | **Female** |
| Ceftrioxone | 31(6.0) | 13(6.7) |
| Ticarcillin/  Clavulanic acid | 256(49.2) | 97(50.0) |
| Ciprofloxacin | 508(97.7) | 191(98.5) |
| Trimethoprim | 424(81.5) | 163(84.0) |
| Cefoperazone/  sulbactam | 431(82.9) | 168(86.6) |
| Cefepime | 501(96.3) | 191(98.5) |
| Amikacin | 245(47.1) | 89(45.9) |
| Gentamicin | 460(88.8) | 179(92.3) |
| Imipenem | 512(98.5) | 193(99.5) |
| Meropenem | 511(98.3) | 194(100) |
